# Prediction of response to drug therapy in psychiatric disorders

**DOI:** 10.1098/rsob.180031

**Published:** 2018-05-23

**Authors:** Shani Stern, Sara Linker, Krishna C. Vadodaria, Maria C. Marchetto, Fred H. Gage

**Affiliations:** Laboratory of Genetics, Salk Institute for Biological Studies, La Jolla, CA 92037, USA

**Keywords:** prediction, classification, bipolar disorder, major depression, autism spectrum disorder, schizophrenia

## Abstract

Personalized medicine has become increasingly relevant to many medical fields, promising more efficient drug therapies and earlier intervention. The development of personalized medicine is coupled with the identification of biomarkers and classification algorithms that help predict the responses of different patients to different drugs. In the last 10 years, the Food and Drug Administration (FDA) has approved several genetically pre-screened drugs labelled as pharmacogenomics in the fields of oncology, pulmonary medicine, gastroenterology, haematology, neurology, rheumatology and even psychiatry. Clinicians have long cautioned that what may appear to be similar patient-reported symptoms may actually arise from different biological causes. With growing populations being diagnosed with different psychiatric conditions, it is critical for scientists and clinicians to develop precision medication tailored to individual conditions. Genome-wide association studies have highlighted the complicated nature of psychiatric disorders such as schizophrenia, bipolar disorder, major depression and autism spectrum disorder. Following these studies, association studies are needed to look for genomic markers of responsiveness to available drugs of individual patients within the population of a specific disorder. In addition to GWAS, the advent of new technologies such as brain imaging, cell reprogramming, sequencing and gene editing has given us the opportunity to look for more biomarkers that characterize a therapeutic response to a drug and to use all these biomarkers for determining treatment options. In this review, we discuss studies that were performed to find biomarkers of responsiveness to different available drugs for four brain disorders: bipolar disorder, schizophrenia, major depression and autism spectrum disorder. We provide recommendations for using an integrated method that will use available techniques for a better prediction of the most suitable drug.

## 1. Introduction

The application of personalized medicine for disease diagnosis and treatment is accelerating, enabling targeted therapies for various illnesses. The FDA has approximately 260 drugs with a label of pharmacogenomics, some of which include specific actions to be taken depending on biomarker information such as gene expression differences and chromosomal abnormalities. Personalized medicine is often called precision medicine or genomic medicine, but personalized medicine should be a more generalized term, because genomics is only one way to pre-determine the best medication for a specific patient with a history, symptoms and disease development that are unique to them. Using a variety of biomarkers, an algorithm weighing all these factors into a treatment decision should direct the physician to the best medicine for that specific patient, because although the symptoms experienced by that patient may seem similar to those reported by others, the cause or origin of a disease may be different. A simple example for how a different medication would be better in different cases presenting with similar symptoms is a viral versus a bacterial throat infection. Both diseases present with a sore throat accompanied by fever, but physicians know how to diagnose the best treatment for each of these cases, and the treatment is different.

When dealing with diseases that are more complex in nature, the diagnostic tools become more complex. Psychiatric disorders have been shown to be polygenic; usually the penetrance of a single variant is low and so is its added effect on the overall phenotype. Therefore, providing precision medication requires more sophisticated algorithms. We live in an era in which computational capabilities double every two years [1]; dealing with this reality, accompanied by the outburst of availability of genomic, neuroimaging and other new techniques such as cell reprogramming and gene editing, it is to be expected that the task of finding the most suitable medication will be based on diagnoses and predictions derived from all of these available techniques.

The prevalence of various psychiatric disorders among the population is estimated to be an astonishing number, about 30–40% [2–4]. It is only quite recently, however, that we started realizing the complexity and heterogeneity of the genetics underlying these disorders [5–11]. For example, genome-wide association studies (GWAS) analyses revealed a large number of single nucleotide polymorphisms (SNPs) with a significant association with disease but low penetrance in a diseased population. It may be that a certain amount of variation needs to accumulate or that these variations act as a network to cause the symptoms that we associate with these disorders. It is interesting to note that, in some psychiatric disorders such as autism, less than 3% of the cases are caused by a small number of rare de novo mutations with a high impact [12,13], such as Smith Magenis syndrome [14] (where a deletion occurs on the short (p) arm of chromosome 17 at a p11.2), Rett syndrome (mutation in MECP2 gene) [15] and fragile X syndrome (inherited or de novo mutations in FMR1 gene) [16]. While all share features typical of autism, the cause of each syndrome is very different, and each has been labelled as a sub-category.

By contrast to genetic heterogeneity in complex disease, there is also the other side of the coin: when the same (or similar) genes cause different disorders, pleiotropy. For example, disrupted in schizophrenia 1 (DISC1), neuregulin 1 (NRG1) and CACNA1C mutations and polymorphisms are known to be associated with schizophrenia, bipolar disorder and major depression [17–20]. Another example is the gene dystrobrevin-binding protein 1 (DTNBP1), which has been implicated in schizophrenia, bipolar disorder and major depression [21–24]. There are additional genes implicated in schizophrenia, bipolar disorder and autism spectrum disorder such as NRXN1, CACNA1C, CACNB2 and CNTNAP2 [25–27]. It is possible that some genes are more vulnerable to environmental stress. It also could be that, in order for a phenotype to show, a network of genes acting together is needed; therefore, each gene by itself is not strongly selected against, and they can be well transmitted without loss of fitness in most cases but, with enough accumulation of mutational burden, disease will occur. The main conclusion when it comes to treatment selection is that the origin of each disorder is so complex that no single treatment is best for these disorders; the whole genetic, epigenetic and environmental background should be taken into account.

The diversity of available drugs today for psychiatric disorders alone shows the difficulty of finding the right treatment for the right patient. The mechanism by which these drugs act is usually not well understood. Each drug carries a long list of side effects, so when an ineffective drug is prescribed, not only is it a waste of time for the patient whose symptoms do not improve, but there are also the side effects that act on other systems, sometimes causing long-lasting and irreversible damage. For schizophrenia, for example, there are over 30 suggested drugs, including antipsychotics [28,29]. While 30% of the patients will not respond to the drugs at all, about 30–40% will have a partial response and approximately 30% of the responders will relapse [30]. For bipolar disorder, lithium is currently considered the first-line treatment, but only 30% of bipolar disorder patients will fully respond to it [31,32]. Other drugs used to treat bipolar disorder include mood stabilizers, antipsychotics, a combination of antidepressants and antipsychotics, and anti-anxiety medication [33–35]. For major depression, around 30–50% of the patients have full remission of symptoms with treatment [36–38]. The treatments include selective serotonin reuptake inhibitors (SSRIs), which are prescribed first (e.g. Prozac, Sertraline), norepinephrine–dopamine reuptake inhibitors such as bupropion, and older classes of antidepressants such as tricyclic antidepressants and monoamine oxidase inhibitors [39]. These antidepressants are sometimes combined with mood stabilizers such as lithium and valproic acid [40]. For autism spectrum disorder, drug treatment is used to reduce irritability and aggression. Two drugs have been approved for treatment in children, risperidone and aripiprazole; other drugs include clozapine, haloperidol and sertraline [41]. Yet other drugs are used to treat attention deficit hyperactivity disorder (ADHD) or sleep disturbance; SSRIs are sometimes used to treat anxiety or depression.

In this review, we summarize studies aiming to find predictive biomarkers for the drug that is the most suitable medication for patients with one of the following psychiatric disorders: bipolar disorder, schizophrenia, autism spectrum disorder and major depression. For each disorder we present previous associations and even prediction attempts of treatment outcome using clinical data, GWAS, neuroimaging and other techniques. Currently, the field of prediction of drug outcome is in its infancy, and much work needs to be done. Developing a good predictor will be extremely rewarding because the burden of these diseases on world finances is very large and, more importantly, finding the right drug for the patient will often allow patients to lead normal lives.

## 2. Prediction of drug treatment for bipolar disorder

The first-line treatment for bipolar disorder is lithium, which has the strongest evidence of long-term relapse prevention [42,43]. Lithium reduces mania episodes by 38% and depression episodes by 28% [44]. Other first-line treatments, with weaker evidence of effectiveness, include lamotrigine, valproate, olanzapine, quetiapine, aripiprazole and risperidone [45]. People with managed bipolar disorder can often regain social and occupational functioning; therefore, finding an effective treatment is very important. Early intervention and providing the right treatment early in the progression of the disease have been shown to be very important to the overall outcome of the patient's psychosocial functioning [46,47]. While lithium works very well for some patients, it has adverse side effects such as long-term renal damage, tremors, oedema and weight gain [48–50]. Therefore, predicting the patient's response to the drug both protects the patient from these side effects and allows the clinician to search for a different drug that will stop the mood episodes as soon as possible.

### 2.1. Clinical data

A number of behavioural studies have tried to predict the outcome of lithium treatment. Calkin *et al*. [51] showed that a lower body mass index (BMI) was correlated with a good outcome for treatment but did not show a prediction performance assessment. Sportiche *et al*. [52] studied a large cohort of 754 patients and showed that mixed episodes and alcohol abuse appeared in more than 20% of patients with a partial response or non-response to lithium (*p* < 0.02), and a family history of bipolar type I was associated with a good response (38% in the good response group and 18% in the non-responsive group, *p* ∼ 0.05); there was also a trend (*p* = 0.06) that bipolar type II was associated with a non-response. Kleindienst *et al*. [53] tried to predict a good outcome according to behavioural aspects. They performed a meta-analysis of 43 previous studies and calculated the correlations of 42 clinical features. The data for each feature were available from hundreds of patients. A significant correlation was found with the following features: (1) an episodic pattern of mania–depression interval (positive correlation); (2) a high age of illness onset (positive correlation); (3) a high number of previous hospitalizations (negative correlations); (4) an episodic pattern of depression–mania interval (negative correlation); and (5) continuous cycling (negative correlation).

More clinical data [54] provided the Temperament Scale of Memphis, Pisa, Paris and San Diego–Autoquestionnaire (TEMPS-A) scores that are associated with lithium responsiveness in 71 bipolar disorder patients. A hyperthymic temperament was significantly positively correlated with a good outcome in response to long-term (5 years) lithium carbonate treatment. Depressive, cyclothymic or anxious temperaments were significantly negatively correlated with responsiveness to lithium treatment. Many more behavioural studies have shown that progression of disease, the pattern at which episodes appear, rapid cycling and the number of depression episodes are significantly associated with lithium response/non-response [54–58]. Therefore, clinical data are important parameters when building a response-to-lithium predictor and they should be combined with newer methods for prediction.

### 2.2. Genetics and GWAS

There seems to be a strong genetic component in the response to a certain drug, and specifically to lithium [59,60]. A few studies showed that a good response to prophylactic lithium is a familial trait [59,61–64], pointing more and more in the direction of genomic association. Several genomic studies were used to correlate specific loci with lithium responsiveness. Turecki *et al*. [65] studied a cohort of 247 individuals from 31 families; 106 were considered affected. They were able to map two specific loci associated with responsiveness to lithium with a low *p*-value. The best association was seen in the locus 15q14 (31.46 cM), which had the strongest association, followed by 7q11.2 (84 cM), 6p23 (42.27 cM) and 22q11.2 (4.06 cM). Another study by Perlis *et al*. [66] reported on two cohorts, one with 1177 bipolar disorder type I and type II patients and another with 359 bipolar disorder type I or II patients. With the first cohort, they mapped one strong association locus on chromosome 10p15 and a few other less strong loci (21q21, 12q22 and 6p21). When comparing the two datasets, five loci showed the same direction of effect (8q22, 3p22, 11q14, 4q32 and 15q26). However, no loci met the threshold for genome-wide association. Another study by Turecki *et al*. [67] on 136 excellent lithium responders, 163 controls and 32 families, ascertained through lithium-responsive bipolar probands, showed that polymorphisms in the gene PLCG1 were at a significantly higher frequency in the lithium responder bipolar disorder cohort. A follow-up study [68] with a Norwegian population had similar findings.

Another association [69] was found with a sample of 52 bipolar disorder patients in the Sardinian population. This study found the strongest associations in four SNPs for rs2811332 (intron 4 of the TMCC1 gene), rs1390913 (192953-bp downstream of the GNPDA2 gene), rs869156 (intron 4 of the RASSF4 gene) and rs11869731 (intron 1 of the ACCN1 gene), with *p*-values ranging from 10^−4^ to 10^−5^. The ACCN1 association remained significant even after enlarging the dataset to 204 patients. ACCN1 encodes for a cation channel that is mainly permeable to Na^+^ and to a lesser extent to Li^+^ and K^+^, and it is largely expressed in neurons. Another SNP was found by Masui *et al*. [70], Asn796Ser, a SNP in the BCR gene, when genotyping 161 bipolar disorder patients. The allele frequency of the Asn796Ser SNP was significantly higher in non-responders.

A very strong association was shown by Chen *et al*. [71]. Using a discovery cohort of 294 bipolar disorder I Han Chinese-descent patients, they performed a GWAS and found two SNPs in the GADL1 gene introns that associated with a good lithium response with *p* = 5.50 × 10^−37^ and *p* = 2.52 × 10^−37^. Repetition in another cohort composed of 100 bipolar disorder patients type I had similar results, with *p* = 9.19 × 10^−15^ in each of the SNPs. Prediction based on these SNPs gave a 93% sensitivity. Interestingly, a follow-up study in an Indian population could not replicate these results [72]. Furthermore, Birnbaum *et al*. [73] reported minimal expression of GADL1 in autopsy brains (including those from bipolar disorder patients) and suggested the need to search for lithium effects on kidney function in patients, where this gene is expressed.

Another strong study by Hou *et al*. [74] included 1162 patients in the first GWAS and 1401 in a second GWAS. They found a few SNPs appearing in both datasets, with the most significant ones occurring on four loci on chromosome 21 with *p*-values in the range of 10^−8^–10^−9^. None of the SNPs were in protein-coding gene areas. Two long non-coding RNAs resided in the area; two of these SNPs were located in an intronic region of a long non-coding RNA and the other two were located between these long non-coding RNAs.

A study of 170 bipolar disorder patients who had been followed for 27 years [75] found that patients carrying the T allele for the rs2314339 SNP were 3.5 times more likely to show no improvement for lithium prophylaxis or to experience worsened symptoms with treatment. In another recent study, the International Consortium on Lithium Genetics *et al*. [76] performed a GWAS on 2586 bipolar disorder patients, approximately 90% of European ancestry and 10% Asian. Using the Alda scale, Duffy *et al*. [77] assessed the outcome response of these patients to lithium treatment. They first built a polygenic score for schizophrenia (PGS) using discovery GWAS outcome estimates from 36 989 schizophrenia patients. They then performed a cross-trait meta-analysis and pathway analysis based on the GWAS of the schizophrenia patients and on treatment response in the bipolar disorder patients. They found that a high polygenic score for schizophrenia was inversely associated (*p* < 0.05) with a good response to lithium treatment. This finding concurs with evidence that bipolar disorder patients who have a family history of schizophrenia show poorer response to lithium compared with those with a family history of bipolar disorder [78].

Polymorphisms related to the serotonin regulatory regions were also found to be associated with a good or poor response to lithium treatment. Serretti *et al*. [79] studied the genetics of 201 patients (167 with bipolar disorder and 34 with major depressive disorder) and showed that the variants in the upstream regulatory region of the serotonin transporter gene (5-HTTLPR) were related to the response of the patient to lithium. Subjects with the s/s variant showed a worse response compared to both l/s and l/l variants. Serretti *et al*. [80] followed up this study with a new cohort of 83 bipolar disorder patients and were able to replicate their findings of the better response of l/s carriers, but could not confirm a poor efficacy in subjects with the s/s genotype. Similar results were obtained by Rybakowski *et al*. [81] with a group of 67 patients; in the lithium non-responders, the genotype s/s and the allele s were significantly more frequent than in excellent and partial responders.

GSK-3*β* polymorphisms have also been shown to play a role in a few studies. Benedetti *et al*. [82] studied the polymorphisms in the promoter of the gene encoding for GSK-3*β*, a known target of lithium [83], in 88 bipolar disorder type I patients. Variants in this gene have already been shown to contribute to the risk for bipolar disorder [84]. Benedetti *et al.* [82] found that patients with a T/T polymorphism improved less than patients with a T/C or a C/C. Follow-up studies did not confirm their results in a different cohort of 89 bipolar disorder patients [85] or in another study [86].

### 2.3. Neuroimaging

Another promising prediction method is imaging. Just recently, Fleck *et al*. [87] used functional magnetic resonance imaging (fMRI) and proton magnetic resonance spectroscopy to perform a prediction of outcome to lithium treatment on a group of 20 bipolar disorder patients. They trained an algorithm called genetic fuzzy tree (GFT), which builds logic statements according to the neuroimaging training data, and they were able to predict on the test data (80% train data, 20% test data) whether the patient would have a good outcome to lithium therapy.

Another interesting imaging predictor was built by Kruger *et al*. [88], who performed a regional cerebral blood flow measurement using positron emission tomography (PET); patients were tested during induced sadness by reading a script describing a sad event from the patient's past. The researchers scanned nine patients who were good lithium responders and nine patients who were good valproate responders. For both the lithium and valproate groups, induced sadness resulted in changes in several areas such as the premotor cortex, dorsal anterior cingulate and anterior insula. Comparison of the change patterns in the lithium responders and valproate responders showed differences in the rostral anterior cingulate and the dorsolateral prefrontal cortex.

### 2.4. Other methods

*Electrophysiology*: using whole-cell patch clamp, we previously built a naive Bayes classifier for prediction of lithium response [89]. We measured the physiology of neurons derived from bipolar disorder patients and characterized them using electrophysiological features. We observed, for example, that bipolar disorder neurons from both lithium responders and lithium non-responders shared a larger fast after-hyperpolarization, which is a property that correlates with excitability in other disorders as well [90]. Interestingly, we found several features that distinguished between the neurons that were derived from lithium responders and lithium non-responders. A naive Bayes classifier was built to predict which of the patients from whom the neurons were derived would respond to lithium therapy. Our classifier trained on electrophysiological features from a training set (from known patients) and then used features from a test set (of an unknown patient) to predict whether the test patient would respond to lithium. This was done recursively, each time taking a different patient as the test set and the remaining patients as the training set. We showed that it was possible to predict with an error rate of approximately 5% which of the patients in our cohort would respond to lithium, using features from five patch-clamped neurons. The low error rate provides great promise for developing techniques that will be easier to implement and will be able to provide a good prediction. Our data and those of others [89,91–93] suggest the presence of two sub-disorders in bipolar disorder with very distinct features, and therefore different and distinguishable pre-treatments.

*Thyroid function*: Cole *et al*. [94] studied a group of 65 bipolar disorder type I patients treated with lithium carbonate (57) or divalproex (3) or both (4) and one patient who was treated with carbamazepine. Forty-four of the patients did not respond to monotherapy and also received an antidepressant in addition to the main mood stabilizer. The thyroid tests included thyroid-stimulating hormone (TSH), thyroxine (T4), triiodothyronine (T3) resin uptake and free thyroxine index (FTI). They found that lower FTI values and higher TSH values were significantly associated with a poorer response to lithium. Conversely, the combination of lower TSH and higher FTI was associated with a markedly more rapid remission of depression.

## 3. Prediction of drug treatment for major depression

Major depression is the most prevalent of psychiatric disorders, posing a heavy burden of disease. SSRIs are the most commonly prescribed class of antidepressants. However, approximately 30–40% of patients fail to respond to SSRI treatment entirely, and in those who do respond, weeks of treatment are required before therapeutic effects can be seen. Owing to a limited understanding of the pathophysiology of the disorder, treatment options follow a trial-and-error strategy. Thus, early prediction of treatment–response status may significantly improve the choice of treatments and shorten the time required for achieving remission.

### 3.1. Clinical data

Using clinical data, Chekroud *et al*. [95] predicted the response to citalopram of patients with depression. They studied 25 behavioural features of their patients, such as employment status, years of education, insomnia measures and suicidal thoughts, and predicted with cross-validation the outcome of 164 patients with an accuracy of approximately 65%. They then used their prediction on another validation of escitalopram treatment group of 151 patients. Their predictor gave an accuracy of approximately 60%. They used this model to further predict the response to a combined treatment of escitalopram and buproprion in 134 patients, with an accuracy of approximately 60%. However, the model failed when they used it on a group of 140 patients treated with venlafaxine–mirtazapine (accuracy of approximately 51%, *p* = 0.5), suggesting that the model is specific for mechanisms that are associated with escitalopram.

Mulder *et al*. [96] studied clinical data from 175 patients with depression treated with fluoxetine or nortriptyline and observed an improvement after six months. They found that behavioural features that were associated with a good response were early response and a lower number of schizoid personality disorder symptoms. A poor response was associated with a higher harm avoidance (HA) score, late response and a higher number of schizoid personality disorder symptoms. Following up their work, Mulder *et al*. [97] assessed 164 of these patients after 18 months. Of the 123 patients who were not depressed at six months, 57 (46%) relapsed. Patients who relapsed were more likely to have a history of recurrent depression, have residual depressive symptoms, have a less sustained response to initial treatment, and have avoidant personality disorder symptoms, schizotypal personality disorder symptoms, higher HA scores and lower self-directedness (SD) scores. Of the 38 patients who were depressed at six months, 13 (34%) recovered after 18 months, but no associated features were found.

### 3.2. GWAS

A GWAS association study was performed by Ising *et al*. [98] on a total of 339 inpatients with major depression (85%) or bipolar disorder (15%), and a further 361 inpatients with depression and 832 outpatients with major depression. They found associations with a few SNPs such as the rs6989467 on 8q22 (early partial response to antidepressants, genotypic model, *p* = 7.6 × 10^−7^) or rs1502174 (dominant-recessive model, *p* = 8.5 × 10^−5^) located in the 3′ flanking region of the EPHB1 gene on 3q22. However, no effect withstood correction for multiple testing. They then pooled the data with another independent cohort and performed another GWAS. The highest association found was for rs1912674 (early partial response to antidepressants, *p* = 8.9 × 10^−7^), located in the region between the AK090788 and PDE10A genes on 6q21. No effect remained significant after correction for multiple testing. Next they performed another GWAS for the last cohort and found SNPs that were common with all three cohorts; none of them withstood correction for multiple testing, but 46 SNPs were in the same direction and significant before correction. When they performed a multi-locus analysis in two of the cohorts, they found a significant association of the number of response alleles (high versus low) to response to treatment. Patients with a comorbid anxiety disorder in combination with a low number of response alleles showed the least favourable outcome. The authors stressed the importance of combining information about multiple genetic factors and of also using clinical features in predicting antidepressant response.

A large study [99] involving 1790 major depression disorder patients of European ancestry could not find a single common genetic polymorphism that could significantly predict response to SSRIs and noradrenaline reuptake inhibitors, and no biological pathways were significantly over-represented in the results. No associations were found in a meta-analysis with another large cohort of 2897 individuals. Polygenic scoring found no convergence among multiple associations among the two cohorts.

A sample of 186 major depression patients received 8 weeks of duloxetine treatment in a study performed by Maciukiewicz *et al*. [100]. They used the MADRS score [101] to categorize the responders and non-responders to the treatment. They performed genome-wide logistic regression to find variants related to duloxetine response and extracted the most promising predictors using LASSO regression [102]. They applied support vector machines (SVMs) to construct models, using ten-fold cross-validation. Their classifier performed significantly better than chance (accuracy *p* > 0.1) for response. For remission, it achieved moderate performance with an accuracy = 0.52 and a sensitivity = 0.58. The authors commented that inclusion of additional non-genetic variables might improve prediction.

Observing genetic data that might be associated with response to citalopram, Garriock *et al*. [103] evaluated 430 198 SNPs for association with antidepressant response and remission: for response, 1491 (608 non-responders; 883 responders) subjects; for remission, 1351 (608 non-responders; 743 responders) subjects. Interestingly, they found significant differences in the ethnicity of responders/non-responders, and remitters/non-remitters. They also found significant differences between the responders and non-responders in clinical measures such as employment status and clinical co-morbidities. Association of genotyping results with response to citalopram gave 39 SNPs and 41 SNPs with *p*-values < 1.0 × 10^−4^ for the response and remission phenotypes, respectively. The top two results for response and remission were the same SNPs, rs6966038 and rs6127921, with *p*-values for response of 4.65 × 10^−7^ and 3.45 × 10^−6^. However, none of the SNPs met a genome-wide threshold for significance.

A large cohort study [104] performing a meta-analysis of three datasets and a total of 2394 subjects was performed looking for genomic association with outcomes of four weeks of treatment with SSRIs using the 17-item Hamilton Rating Scale for Depression (HRSD-17) score. No associations were found to be significant at the genome-wide level. The top association to SSRI response found included SNPs in the HPRTP4 (hypoxanthine phosphoribosyltransferase pseudogene 4) and VSTM5 (V-set and transmembrane domain-containing 5) region, which approached genome-wide significance (*p* = 5.03 × 10^−8^), and SNPs 5′ upstream of the neuregulin-1 gene (*p* = 1.2 × 10^−6^). The top associations that were previously published [105,106] with two of the three datasets were not reproduced.

### 3.3. Imaging

A study showing a very strong correlation between neuroimaging by fMRI and the outcome of drug response in major depression disorder was performed by Kozel *et al*. [107] on a cohort of 17 patients. The authors found that connectivity of both subcallosal cortices to the left anterior cingulate gyrus was strongly correlated with outcome of treatment with the antidepressant. The authors commented that the subcallosal cortical region had been shown to be implicated in outcome of treatment with drugs and other types of treatment. The cohort was not very large, and more work should follow with a larger sample size. Generally fMRI is well tolerated by patients, but it is still a relatively expensive form of treatment, which should be taken into consideration.

Using PET, McGrath *et al*. [108] looked for predictors of the response to escitalopram or behavioural therapy in 38 major depression disorder patients. Right anterior insula normalized metabolism was shown to have the most significant correlation. Insula hypometabolism was associated with remission of symptoms after cognitive behaviour therapy and poor response to escitalopram, whereas insula hypermetabolism was associated with remission of symptoms after escitalopram treatment and poor response to cognitive behaviour therapy.

Gong *et al*. [109] used high-field MRI and built an SVM classifier. Sixty-one patients and 42 healthy volunteers were scanned using structural magnetic resonance imaging. Patients then received standard antidepressant medication (tricyclic, typical serotonin–norepinephrine reuptake inhibitor or typical SSRI). SVM applied to grey matter images correctly distinguished between responders (23) and non-responders (23) with an accuracy of approximately 70% (*p* = 0.006). SVM applied to white matter images predicted clinical outcome with an accuracy of approximately 65% (*p* = 0.02).

In a large meta-analysis, Fu *et al*. [110] analysed 20 studies for functional neuroimaging (MRI and PET) of 15 independent samples and nine studies on a different set of six independent samples for structural MRI. Using functional neuroimaging, they found a significantly increased activation associated with higher likelihood of good response to treatment in the anterior cingulate and medial prefrontal cortices, with a cluster in the left pregenual anterior cingulate and smaller clusters in the right pregenual anterior and right subgenual cingulate/medial orbitofrontal cortex. For the amygdala, some reports were of increased activity and some of decreased activity associated with a positive therapeutic outcome. In the anterior cingulate, a few studies reported that greater baseline activation in the subgenual region was predictive of a poorer response. In the structural studies, voxel-based morphometry showed an association between a decrease in grey matter volume in the dorsolateral prefrontal cortex [109,111–113] and a poor response to antidepressants, but this finding was insignificant after false discovery rate multiple comparisons correction. A significant association was revealed between decreased right hippocampal volume and lower likelihood of benefit from treatment (*p* = 0.0038). One of the studies also linked reduced response with lower caudate nucleus volumes [114].

Little *et al*. [115] used fluorine-18 deoxyglucose PET as a biomarker for six weeks of bupropion or venlafaxine monotherapy on never-hospitalized patients with unipolar depression. They found that, compared with control subjects, responders (*n* = 11) but not non-responders (*n* = 9) to both drugs demonstrated frontal and left temporal hypometabolism. Bupropion responders (*n* = 6) also had cerebellar hypermetabolism compared with controls, whereas venlafaxine responders (*n* = 7) showed bilateral temporal and basal ganglia hypometabolism compared with controls.

### 3.4. Other methods

*Electroencephalography* (EEG): Bares *et al*. [116] predicted the treatment outcome of several antidepressants on a cohort of 87 patients with similar baseline clinical conditions. They used three parameters to predict the outcome of the response after five weeks: (1) reduction of prefrontal theta cordance value measured by EEG; (2) reduction of symptoms according to the Montgomery and Åsberg Depression Rating Scale after one week; and (3) reduction of the symptoms after two weeks. A combination of these three parameters resulted in an area under the curve (AUC) of 0.91 using the receiver operating characteristics (ROC). This is a strong result with a reasonable cohort size; therefore, EEG measurements may be another promising direction for prediction of treatment.

## 4. Prediction of drug treatment for schizophrenia and schizoaffective disorder

A large meta-analysis [117] with 65 trials involving 6493 patients showed the superiority of treatment with antipsychotics compared with placebo. The antipsychotics reduced the relapse rate to less than half and readmission rates to less than half. Treatment needed to be maintained, and relapse rates did not change after a few years of treatment. Outcome of treatment is a further good predictor of whether a patient will have a functional impairment after the hospitalization period [118]. Specifically, positive and negative symptoms during the drug treatment period were good predictors of future functioning of the patient, and symptoms during the drug-free period were not. Therefore finding and maintaining the right treatment is crucial for better functioning of schizophrenia patients.

### 4.1. Clinical data

Kinon *et al*. [119] tested whether an early good response to risperidone (two weeks) predicted the response at 12 weeks with a large cohort of 628 patients diagnosed with schizophrenia or schizoaffective disorder. The early responders had a significantly better outcome after 12 weeks, and the non-responders benefited significantly from switching to olanzapine. Similar results were obtained by Correll *et al*. [120], with 95 patients with acute schizophrenia after four weeks of fluphenazine treatment. They observed that the patients who had a bigger improvement after one week were also more likely to have an improvement after four weeks, with approximately 70% predictive power. Kinon *et al*. [121] also looked at how an early response predicted a later response. They studied 1077 moderately affected schizophrenia patients (84% diagnosed with schizophrenia and the rest with schizophreniform, or schizoaffective disorder). A total of 325 were early responders. The study predicted according to the response at two weeks what the response would be at three months, with an AUC of 0.75.

Similarly, Stauffer *et al*. [122] looked at the early response as a predictor of the later response to olanzapine or haloperidol in 168 schizophrenia patients with their first psychotic episode. The early response at two weeks predicted the later response at 12 weeks with approximately 74% specificity and approximately 80% negative predictive value. Another study showing this early prediction phenomenon [123] examined response to ziprasidone or olanzapine in 94 schizophrenia or schizoaffective disorder patients. Improvement at two weeks predicted further improvement at six months with an AUC of 0.85.

Kohler-Forsberg *et al*. [124] sought possible predictors of the outcome of clozapine treatment in 502 clozapine-treated schizophrenia patients. The main predictor of a good outcome in their study was living with a partner.

### 4.2. Genome-wide association studies

Arranz *et al*. [125] performed a study on 200 schizophrenia patients (133 patients were classified as responders and 67 as non-responders) and looked for associations with outcome of drug response. They found 19 polymorphisms with prediction power of the drug response. The prediction levels were calculated by logistic-regression analysis, with the response to clozapine as the dependent variable and the polymorphisms studied as independent variables. The positive predictive value for these results was 0.76, and the negative predictive value was 0.82.

Reynolds *et al*. [126] performed a GWAS for 117 schizophrenic Chinese Han patients who were first episode and drug-naive, followed by a 10-week antipsychotic treatment, primarily with risperidone or chlorpromazine. They specifically looked at the dopamine D3 receptor ser9gly, the dopamine D2 receptor Taq IA and the 5-HT2C receptor promoter—759C/T polymorphisms. The D3 receptor ser9gly polymorphism was significantly associated with improved symptoms and also associated with initial behavioural symptoms on admission. The 5-HT2C receptor 759C/T was also associated with improvement after treatment but was not associated with a baseline score.

McClay *et al*. [127] genotyped 738 patients with schizophrenia and looked for associations with response to treatment with the antipsychotics olanzapine, quetiapine, risperidone, ziprasidone and perphenazine. One SNP passed their threshold criterion of less than 10% false discovery; it was located in an intergenic region on chromosome 4p15. In addition, SNPs in ankyrin repeat and sterile alpha motif domain-containing protein 1B (*ANKS1B*) and in the contactin-associated protein-like 5 gene (*CNTNAP5*) were very close to the threshold.

Another study [127] with 738 subjects with schizophrenia looked at genetic variation underlying individual differences in response to treatment with the antipsychotics olanzapine, quetiapine, risperidone, ziprasidone and perphenazine. The top finding was a SNP rs17390445 on chromosome 4p15, which mediated the effect of ziprasidone on positive symptoms with a *q* value of a little less than 0.05 (*p* = 9.8 × 10^−8^). Another SNP rs11722719, residing 1.6 kb from this SNP, had a *q*-value of less than 0.15 (*p* = 5.4 × 10^−7^) and it also mediated ziprasidone-positive symptoms. SNP rs7968606 in the *ANKS1B* gene showed a *q*-value of 0.16 (*p* = 3.2 × 10^−7^) for mediating the effect of olanzapine on negative symptoms. The finding for SNP rs17727261 in the *CNTNAP5* gene, mediating the effects of risperidone on negative symptoms, had a *q*-value of 0.13 (*p* = 5.4 × 10^−7^). Another interesting SNP, rs17815774 in the *TRPM1* gene, mediated the effects of risperidone on negative symptoms, with a *q*-value of 0.4 (*p* = 3.3 × 10^−6^).

Zhang *et al*. [128] looked at the DRD2 (dopamine D2 receptor) locus that was associated with risk of schizophrenia by a large-scale GWAS from the Psychiatric Genomics Consortium [129]. They studied whether the SNP rs2514218 could predict an antipsychotic response in a cohort of patients with a first episode of psychosis treated with either risperidone or aripiprazole for 12 weeks. Data were collected from 100 subjects, approximately half treated with risperidone and half with aripiprazole. Linear mixed model analysis showed that the homozygotes for the risk (C) allele had significantly greater reduction in positive symptoms during 12 weeks of treatment compared with the T allele carriers (*p* = 0.044). The DRD2 gene has previously been shown to be associated with responsiveness to antipsychotics. Ikeda *et al*. [130] also showed that a SNP in DRD2 was a significant predictor of the response to risperidone along with a SNP in TaqIA and two SNPs in AKT1.

### 4.3. Imaging

Kapur *et al*. [131] have studied the response to haloperidol of 22 patients with moderate to severe symptoms. Of these, 21 were classified with schizophrenia and one with delusional disorder. The responders showed significantly higher dopamine D2 receptor occupancy, as determined with raclopride and PET [132], after two weeks of treatment (mean = 73%, s.d. = 9%) than the non-responders (mean = 60%, s.d. = 12%), *p* < 0.009. Using it as a predictor with a cutoff at 65%, D2 occupancy provided optimal separation: 80% of the responders were above it whereas 67% of the non-responders were below it (*p* = 0.04, Fisher's exact test).

## 5. Prediction of drug treatment for autism spectrum disorder

Only two drugs (risperidone and aripiprazole) have been approved by the FDA for treatment of children with autism spectrum disorder, and these two drugs mainly target irritability. The ability to predict a benefit from drug treatment is very important in autism spectrum disorder, because the patients are often young children, and the total effects of drug treatment on their development are often hard to foresee. Very little has been done in terms of predicting positive outcomes from drugs; however, a few studies suggest the association of clinical data and GWAS with drug treatment outcome.

### 5.1. Clinical data

Using the aberrant behaviour checklist (ABC) [133] irritability test subscale score, Arnold *et al*. [134] have observed several moderators and mediators that may affect the outcome of the response to risperidone. In the moderator list, a high baseline ABC irritability subscale was associated with a larger decrease in irritability compared with a low baseline. Other predictors showing the association of a good outcome included a higher parent education level, a higher income of parents and a lower baseline level of prolactin, but these were correlated with an improvement both in the drug treatment and the placebo groups. A negative association was found with higher levels of anxiety, an accompanying bipolar disorder, oppositional defiant symptoms (hostile, disobedient and defiant behaviours), high hyperactivity and high stereotypic behaviour. In the mediator group, a weight gain with treatment was negatively correlated with a decrease in irritability but it was positively correlated with a decrease in irritability in the placebo group. The dose of risperidone was also positively correlated with a decrease in irritability.

### 5.2. Genome-wide association studies

Correia *et al*. [135] performed a study on 31 children with autism spectrum disorder after a one-year treatment with risperidone, and their improvement was assessed by the Autism Treatment Evaluation Checklist (ATEC). A few polymorphisms were found to be associated with improvement. The *HTR2A* c.-1438G > A, *DRD3*Ser9Gly, *HTR2C* c.995G > A and ABCB1 1236C > T polymorphisms were predictors of clinical improvement with risperidone therapy. The *HTR2A* c.-1438G > A, *HTR2C* c.68G > C, *HTR6* c.7154–2542C > T and *BDNF* c.196G > A polymorphisms influenced prolactin elevation. *HTR2C* c.68G > C and *CYP2D6* polymorphisms were associated with a risperidone-induced increase in BMI or waist circumference.

### 5.3. Transcriptome

Lit *et al*. [136] studied peripheral blood gene expression in 42 patients with autism spectrum disorder before treatment with risperidone and then looked at the eight-week treatment outcome. They identified 89 exons that have a strong association with treatment outcome and separate responders from non-responders using unsupervised hierarchical cluster analysis. Among the genes were GBP6, RABL5, RNF213, NFKBID and RNF40.

## 6. Discussion

The field of precision medicine for psychiatric disorders is in its initial phase. Accurate prediction attempts of treatment efficacy are rare. There are, however, quite a few studies showing associations and correlations of different biomarkers with a good outcome of drug treatment. The methods used include neuroimaging, electrophysiology, GWAS studies, behavioural studies, animal models, EEG, transcriptomics and others. Importantly, to accomplish the task of assigning the best drug to each patient, the studies that are most relevant are those that predict outcomes of specific drugs. To build precision medication tools, it is important to identify the features that will direct the clinician to the drug that is most suitable for a specific patient.

Building a large dataset requires the repository of data from thousands of people. Ethical questions such as who would have access to these data or tests required by insurance companies should be addressed [137,138]. An important question that needs to be asked when getting a suggestion of treatment from a computer program is: what is the minimal accuracy that we should request before prescribing a patient with a drug? This is probably where the physician's experience and knowledge would be useful. Medicine with adverse side effects should be given only if the error rate of the prediction is very low, and also then monitored closely. With medicine with fewer side effects, the accuracy requested from the algorithm can be relaxed. A good algorithm should also give multiple treatment options with a list of preferences.

Reviewing previous studies of associations between drug response and genomic and biological markers, a major downside that is very evident is the lack of replication. Work needs to be repeated, sometimes in a larger cohort, to make sure that these markers for drug response outcomes are reproducible. Despite the fact that a repeated study makes smaller headlines, it is our responsibility to check for reproducibility and robustness. Markers that were found to robustly associate with a good drug response can then be used for robust predictions.

**Figure 1.**
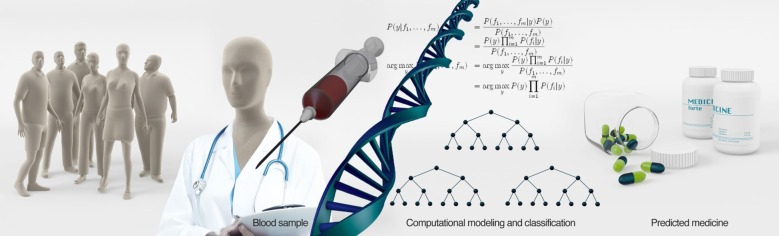
Recent studies are focusing on finding genomic markers for predicting the outcome of treatment using specific drugs. A simple blood test can be used for DNA sequencing. Prediction based on DNA sequencing shows great promise, and there are quite a few recent studies using this technique. However, this technique alone may be insufficient for an excellent prediction of drug outcome and should be accompanied by other methods.

A relatively large amount of work has been done in GWAS, with quite a few associations. The genomic revolution now allows for fast and cheap DNA sequencing, and may bring great promise to the prediction of optimal treatment (figure 1). But are these predictions good enough, and do they have a high enough prediction power? The path from variants and SNPs to neuronal function is long and mainly unresolved. SNPs and variants work very well with the cell machinery, but can a computer program imitate the cell's ability to know how these small changes to the DNA sequence would translate into amounts of RNA and proteins, and eventually a phenotype? A study that shows a response in the function of neurons [89,139] provides a lot of information that can be used when building a predictor, because scientists can now associate variants with drug response with a much higher likelihood, even if this variant is not seen in larger populations. It is therefore worthwhile to work backwards from functional assays such as electrophysiology to identify biomarkers that correlate with the functional measurements. Common genetic features that associate with these biomarkers can then be identified and used for predictions.

It is also important to note that different genomic changes may lead to similar results. In performing GWAS with a large number of patients, different genomic variants that converge to a similar phenotype may be lost in the analysis and appear insignificant. While each of these genomic changes may appear in a small population of the disease, they may all eventually cause similar phenotypes (different small genomic changes converging to the same phenotype). We hypothesize that RNA expression may be more closely and immediately associated with the final phenotype; therefore, looking at RNA expression or protein levels will give us much better clues as to how cell function will be altered by genomic changes. One problem is that we collect blood samples from the patients, not neurons, which is where the disease is expected to manifest. The use of induced pluripotent stem cells to study disease allows for differentiation followed by transcriptomics, but this is a long process. The question is whether any of these transcriptomic changes could be observed in blood cells. Epigenomics may give us the answer, because genes that are repressed may be in a more compact area of the chromatin. We believe that more transcriptomic and epigenomic studies should be performed to identify changes that play a role in the response to specific drugs to complement the large number of GWAS studies.

**Figure 2.**
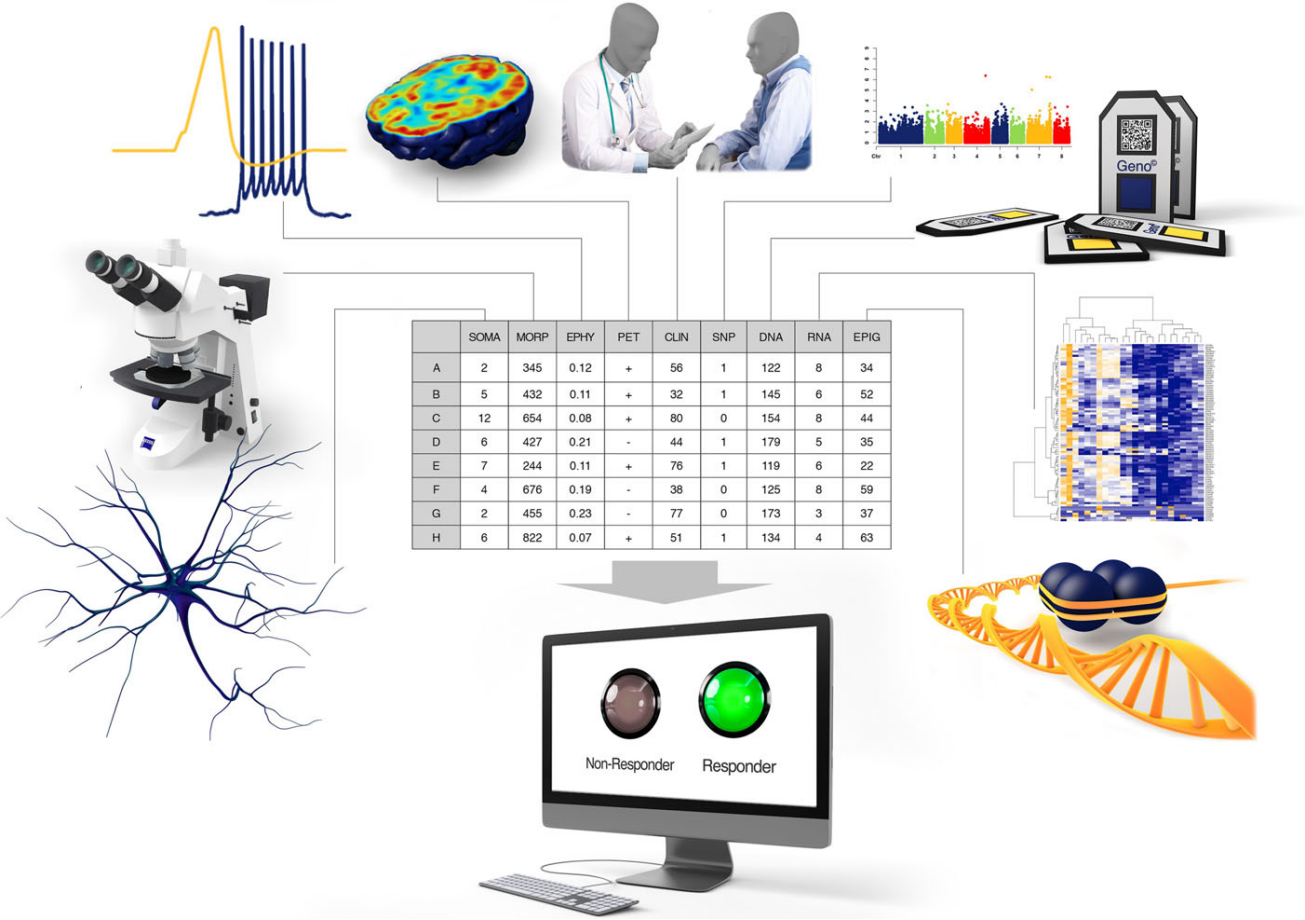
Using multiple available techniques may improve prediction greatly. We have reviewed here studies aiming at predicting the right treatment using morphology, electrophysiology, imaging, genomics, transcriptomics, epigenomics and clinical data. The ultimate classifier should incrementally add features from different techniques. Using cross-validation, the classifier would conditionally add a new feature to the training set, and then check whether this new feature improves the prediction on the test set. After cross-validating the entire dataset, only if a new feature indeed improves prediction results would it be added permanently to the feature set for prediction. This way the classifier would use features extracted from multiple methods, weigh them and provide an optimal prediction based on features that improve its performance.

Importantly, when building classification algorithms, features extracted from different methods can be used together for prediction, and an algorithm does not need to be restricted to a specific method (figure 2). The methods should be easy to implement in order to minimize the time needed to find the right treatment, and they should preferably not be very expensive. For example, sequencing of blood in combination with behavioural assessment is easy, and may provide a quick and relatively inexpensive prediction. Figure 2 shows a general plot of an algorithm that would use features extracted from measurements using different methods for a prediction of the best drug. Much work needs to be put into finding these biomarkers that distinguish populations and make patients drug-responsive or non-responsive. The computational power exists and so do the tools, and we need to use this opportunity to develop tools to help psychiatric patients and their families regain their quality of life.

## Data accessibility

This article has no additional data.
